# Polymeric-Micelle-Based Delivery Systems for Nucleic Acids

**DOI:** 10.3390/pharmaceutics15082021

**Published:** 2023-07-26

**Authors:** Genada Sinani, Meltem Ezgi Durgun, Erdal Cevher, Yıldız Özsoy

**Affiliations:** 1 Department of Pharmaceutical Technology, Faculty of Pharmacy, Altinbas University, 34147 Istanbul, Türkiye; genada.sinani@altinbas.edu.tr; 2 Department of Pharmaceutical Technology, Faculty of Pharmacy, Istanbul University, 34126 Istanbul, Türkiye; mezgi.kilic@istanbul.edu.tr (M.E.D.); ecevher@istanbul.edu.tr (E.C.)

**Keywords:** polymeric micelle, micelleplex, polyplex, polyion complex micelle, cationic polymer, gene delivery, nucleic acid, DNA, RNA

## Abstract

Nucleic acids can modulate gene expression specifically. They are increasingly being utilized and show huge potential for the prevention or treatment of various diseases. However, the clinical translation of nucleic acids faces many challenges due to their rapid clearance after administration, low stability in physiological fluids and limited cellular uptake, which is associated with an inability to reach the intracellular target site and poor efficacy. For many years, tremendous efforts have been made to design appropriate delivery systems that enable the safe and effective delivery of nucleic acids at the target site to achieve high therapeutic outcomes. Among the different delivery platforms investigated, polymeric micelles have emerged as suitable delivery vehicles due to the versatility of their structures and the possibility to tailor their composition for overcoming extracellular and intracellular barriers, thus enhancing therapeutic efficacy. Many strategies, such as the addition of stimuli-sensitive groups or specific ligands, can be used to facilitate the delivery of various nucleic acids and improve targeting and accumulation at the site of action while protecting nucleic acids from degradation and promoting their cellular uptake. Furthermore, polymeric micelles can be used to deliver both chemotherapeutic drugs and nucleic acid therapeutics simultaneously to achieve synergistic combination treatment. This review focuses on the design approaches and current developments in polymeric micelles for the delivery of nucleic acids. The different preparation methods and characteristic features of polymeric micelles are covered. The current state of the art of polymeric micelles as carriers for nucleic acids is discussed while highlighting the delivery challenges of nucleic acids and how to overcome them and how to improve the safety and efficacy of nucleic acids after local or systemic administration.

## 1. Introduction

Advances in biology, medicine and genetics have led today to a better understanding of diseases and their genetic nature. These advances have also given rise to innovations in drug research and development as well as delivery technologies for therapeutics.

Consecutively, the idea of treating the underlying factor of the disease by modulating disease-causing gene activity instead of by using the classical approach of treating the symptoms of the disease with conventional drugs has started to revolutionize healthcare. This gene-based therapeutic approach, known as gene therapy, can be used to treat not only diseases caused by genetic disorders but also acquired diseases such as infectious diseases or cancer [1]. Gene therapy, using, among other treatments, plasmid DNA (pDNA), messenger RNA (mRNA), antisense oligonucleotides (ASOs) and small interfering RNA (siRNA), has shown great potential compared to conventional therapies and shows huge potential to be used in clinic. However, the clinical translation of nucleic acids is challenging because of their hydrophilicity, high molecular weight, fragile structures susceptible to in vivo degradation, and low stability, as well as the difficulty involved in reaching the site of action. Thus, they require a delivery system that can safely and efficiently transport them to the target site [2].

Indeed, the determination of the nucleic acid carrier vector is the most important point when designing gene delivery systems. In the literature, carrier vectors used for gene therapy are classified into two main categories: viral and non-viral. As the name indicates, viral systems consist of viruses in their non-pathogenic form, which have been modified to be replication-deficient. Adenoviruses, adenovirus-associated viruses, retroviruses, lentiviruses and bacteriophages are widely used as viral gene delivery vectors. However, viral vectors show disadvantages in terms of the induction of unwanted immune responses and are characterized by limited packaging capacity [3,4].

Non-viral gene delivery systems have been developed as an alternative to viral systems. The most important advantages of these systems are their enhanced safety profile and their large packaging capacity for genetic material [5]. Non-viral methods for gene transfer are divided into physical and chemical subgroups. Physical methods consist of gene gun, microinjection via needles, laser irradiation, electroporation and sonoporation techniques [6]. On the other hand, chemical methods can be categorized as organic and inorganic. The organic category includes carrier systems such as polymeric micelles, dendrimers and liposomes [7], while the inorganic category consists of delivery systems such as the silver nanoparticles or magnetic nanoparticles of inorganic materials [6].

**Figure 1:**
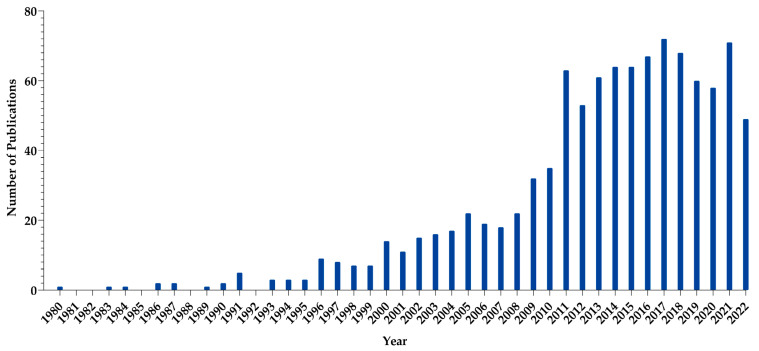
Micelle-related articles published from 1980 to 2022. (Data gathered from PubMed using the keywords micelle, plasmid DNA, antisense nucleotide, mRNA and siRNA).

Micelles are widely used as non-viral systems in gene therapy [8]. Biocompatibility, ease of preparation, the opportunity to adjust particle size, good long-term stability and the possibility to control their physicochemical characteristics are the most important factors in the preference of micelles [9]. The history of using micelles as a carrier system in gene therapy goes back to the 1980s. Since the first years of its use, micelles have attracted increasing attention as versatile delivery vehicles for nucleic acids. Figure 1 displays the PubMed database of studies related to micelle-based systems developed for the purpose of gene therapy [10].

Micelles are commonly defined as self-assembled structures of amphiphilic molecules above a specific minimal concentration known as the critical micelle concentration (CMC). While micelles are characterized by a hydrophobic inner core and a hydrophilic outer shell, structures named “reverse micelles” or “inverse micelles’’ consisting of a hydrophilic core and a hydrophobic shell can also be formed [11]. Although the term “micelle” is widely used in the literature to indicate, in general, the formation of core–shell micellar structures, polymeric micelles, specifically, are nano-sized carrier systems that are formed by the self-assembly of amphiphilic blocks (di- or tri-) or graft polymers above the CMC. Polymeric micelles can incorporate and transport the lipophilic agents in the hydrophobic inner core and hydrophilic agents in the hydrophilic outer shell. Compared to micellar structures composed of conventional surfactant molecules, i.e., small molar mass molecules, polymeric micelles are formed at lower CMC values and tend to be more stable in vitro and in vivo [12,13]. For this reason, polymeric micelles are widely used in studies as nanocarrier systems for the delivery of drugs and other therapeutic agents.

The polymers used in the preparation of polymeric micelles can be natural or synthetic [14]. Owing to the development of polymerization technology, copolymers with different structures and desired physicochemical properties can be synthesized using various hydrophilic, hydrophobic and cationic blocks [8,15]. Thus, the structural properties of polymeric micelles, such as their surface charge and size and the release mechanism of the therapeutics, can be altered to design effective delivery systems and optimize other factors, such as cellular uptake, endosomal escape and cellular release, that influence treatment efficacy [13]. Furthermore, polymeric micelles offer the opportunity to delivery both drugs and nucleic acid therapeutics simultaneously. Therefore, they have been gaining increased interest to achieve synergistic combination treatment particularly with targeted cancer treatment [16]. These advantages have made polymeric micelles an important non-viral delivery system for nucleic acids (NA).

## 2. Polymeric Micelles

**Figure 2:**
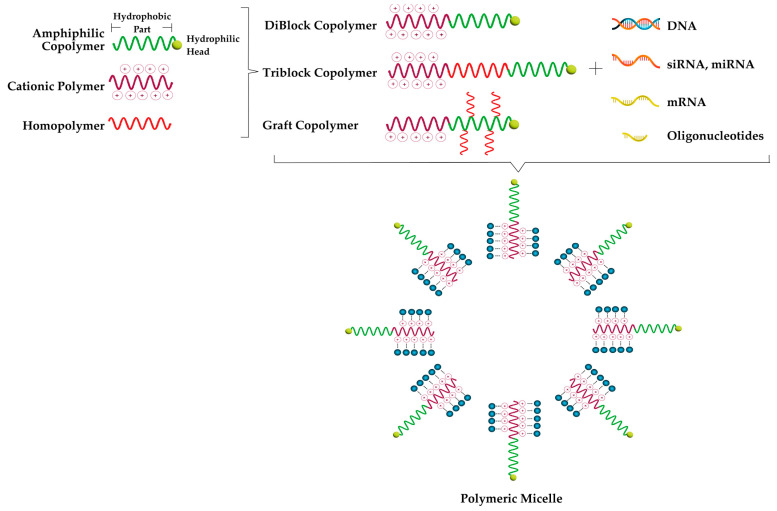
Copolymer type and formation of polymeric micelles.

Polymeric micelles (PMs) are generally 10–100 nm in size and consist of copolymers containing hydrophilic and hydrophobic blocks [17] (Figure 2). Copolymers can be amphiphilic di-block, tri-block or graft structures. While polyethylene glycol (PEG)/polyethylene oxide (PEO), polyvinyl alcohol (PVA), polyvinyl pyrrolidone (PVP), polyacrylic acid (PAA), polyacrylamide (PAAm) or polyglycerol (PG) are often preferred to form hydrophilic blocks of copolymers, polyesters (poly(caprolactone) (PCL), poly(d,l-lactic acid) (PDLLA) and poly (glycolic acid) (PGA)) or polyethers, e.g., polypropylene oxide (PPO), can be used to form hydrophobic blocks [18,19].

Nucleic acids characterized by an extensive negative charge electrostatically interact with micelle-forming charged polymers and form stable nucleic acid–polymer complexes [20]. These are generally referred to as polyion complex (PIC) micelles in the literature. PIC micelles were first introduced in 1995 by Kataoka et al. [21]. In their study, they showed that the formation of a micelle structure was based on electrostatic interactions between copolymers carrying oppositely charged and neutral hydrophilic segments, unlike what was known up to that time. In the following years, different groups working in this field have similarly developed polymeric micelles formed by electrostatic interaction. However, each group gave a different name to the structure formed. Block ionomer complexes (BIC) [22], complex coacervation core micelles (C3Ms) [23], or (inter)polyelectrolyte complexes (I)PEC [24,25] are examples of these names.

Unlike classical micelles, the core of PIC micelles consists of a polyion complex. Their shells are composed of segments of neutral copolymers. The most important advantage of PIC micelles is their spontaneous formation in an aqueous solution under thermodynamic equilibrium conditions [26]. Various hydrophilic macromolecules such as peptides, proteins, nucleic acids and oligonucleotides, as well as multivalent block copolymers as their oppositely charged counterparts, can form PIC micelles via electrostatic interaction. These self-assembly structures formed between copolymers, DNA and RNA can be modified to achieve greater specific activity for nucleic acid cargo and can also be referred to as polyplexes [27] or micelleplex [28]. However, they are called PM or PIC micelles in the continuation of this review.

Each of the hydrophilic, cationic and hydrophobic segments of the copolymers used in the production of PIC micelles affects their characteristic properties. Parameters such as size, surface characteristic, release of the therapeutic agent, and NA binding, which are discussed in detail in Section 2.2 of this review, directly affect their efficacy and stability [8]. Developments in polymerization techniques that offer the possibility to functionalize polymers by using reactive monomers, the chemical modification of end-groups, and the inclusion of degradable links or cross-linking give the opportunity to engineer the structure of PIC micelles [29]. Consecutively, the in vivo behavior of PIC micelles such as cellular uptake and endosomal release can be improved, and therapeutic efficacy can be increased.

### 2.1. Preparation of Polymeric Micelles

Various methods have been reported in the literature for the preparation of PMs. The selection of the preparation method depends on the physicochemical characteristics of the polymer and the therapeutic agent, i.e., a drug or a nucleic acid. Two steps can be involved in the preparation of NA-carrying PMs. Firstly, the PM structure is formed [9]. This is followed by the incorporation of NAs to the PM [8]. Hence, describing the preparation of PMs briefly under two main subheadings, as follows, would be a correct approach for this review.

#### 2.1.1. Preparation Methods of PMs

- Direct Dissolution Method

The direct dissolution method is the simplest method among all other methods. PMs are formed by a mixing therapeutic agent and polymer molecules at the CMC or at higher concentrations in an aqueous medium. It is based on the principle that micelles form completely by themselves. The main disadvantage of this method is that the drug loading capacity is relatively low [30].

- Dialysis Method

This method is based on replacing the organic solvent in the dialysis bag with water over time. The organic solvent used must be miscible with water. The drug and polymer are dissolved in the organic solvent and placed in the dialysis bag, and dialysis takes place against the water [31]. This method has two major drawbacks [32]. The first is that removing all micelles from the dialysis bag after production is not easy. The second is that it cannot be appropriate for scale-up and is only suitable for laboratory-scale productions [30].

- Oil-in-Water (O/W) Emulsion

First, the drug and/or polymer is dissolved in an organic solvent that is immiscible with water. Then, this solution is mixed with water until a homogeneous emulsion is obtained. The organic solvent in the resulting emulsion is evaporated to obtain micelles in the aqueous phase [30,33,34,35,36].

- Thin Film Layer (Solvent Evaporation)

After the drug and polymer are dissolved in a volatile organic solvent, organic solvent is evaporated. The evaporation of the solvent creates a thin drug–polymer film around the flask [32,37,38]. This film layer is then hydrated with an aqueous phase. The most important advantage of this method is that it can be applied quickly and easily [30].

- Lyophilization Method

After the drug and polymer are dissolved in an organic solvent suitable for lyophilization, they are lyophilized by mixing with water. Lyophilized micelles are then applied by dissolving them in an isotonic aqueous medium or sterile distilled water [39,40]. The most important advantage of this method is that it is suitable for heat sensitive drugs and sterile large-scale productions. Nevertheless, the necessity to use organic solvents suitable for lyophilization and the risk of residual solvents are drawbacks for this method [30].

#### 2.1.2. Preparation of Nucleic Acid-Carrying PMs

- Conjugation

Conjugation is a method developed based on bioconjugation technology, which affects almost all disciplines of life sciences. In bioconjugation, two or more molecules bind to form a new complex, and this new molecule has the properties of both of the molecules that make it up [41]. Based on this basic information, it was predicted that NA could be directly conjugated to the PM core [8]. In this method, NAs form covalent bonds with polymer or lipidic blocks. The conjugation of NA with polymers can enhance the pharmacokinetic profile of NA, e.g., provide advantage over enzymatic degradation, instability in physiological conditions or rapid renal clearance [42].

PIC micelles, in which NAs are conjugated with polymers, have been widely obtained using the thin film layer preparation method. In these studies, after the preparation of the polymer solution in an organic solvent, the organic solvent was evaporated [43]. Then, aqueous solution containing the NA–polymer conjugate was used in the hydration process. There have been many conjugation-based studies of si-RNAs [43,44,45,46,47,48], mi-R145 [49] and miRNA-34a-ss [50] with different polymers. However, the choice of the organic solvent used during conjugation is very important in terms of the reproducibility and prevention of NA denaturation [51]. To avoid this, NA–polymer binding can also be performed using the direct dissolution method. In this case, stability problems can be observed in the PIC micelles under physiological conditions [8].

- Rolling circle amplification (RCA)-assisted

Studies with this method are based on DNA [52,53,54]. There are different studies showing that a stable PM is produced with hydrophobic polymers conjugated with single-stranded (ss) DNA. In the studies, firstly, a polymer–DNA primer was synthesized and then ss DNA was produced via RCA. This method also allows the generated DNA RCA products to be hybridized later with oligonucleotides such as siRNA [52].

- Complexation

Complex formation between the polymer and NA by electrostatic interactions is based on the formation of an NA–polymer structure by the direct dissolution method, resulting in the formation of PIC micelles [8]. If the polymer is not soluble in water, the dialysis method can also be applied by using a suitable organic solvent. It was observed that the mixing order of polymers and NA affected the micelle size and PDI when the direct dissolution method was used [55]. Thus, each step must be investigated in detail, and the whole process must be carefully optimized to ensure the repeatability of production and scale-up production.

- Core Loading

In the core loading method, NAs are simultaneously encapsulated in the core of the micelles [56]. Liang et al. prepared micelles by the O/W emulsion method. First, the doxorubicin-loaded micelles were prepared using chloroform, and then miR-519c was added to the medium to prepare a water-in-oil (W/O) emulsion. Then, by adding water to this emulsion as an external phase, a water/oil/water phase emulsion was obtained.

**Figure 3:**
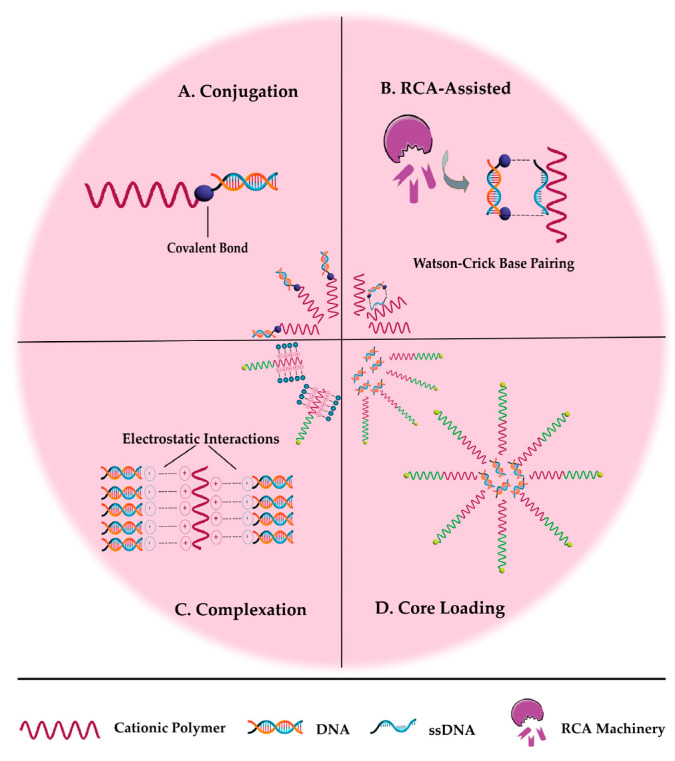
Polymeric micelles carrying nucleic acids.

The approaches described above used to prepare PMs carrying NAs are illustrated in Figure 3.

### 2.2. Characteristic Features and Characterization Methods of Polymeric Micelles

#### 2.2.1. Size, Size Distribution, Surface Characteristics and Morphology

For PM size, PDI, surface charge and morphological features are the most basic characteristic features. PM sizes are generally expected to be between 10 and 100 nm [17]. The size has great importance for their delivery to the target area. Considering that micelles are generally transmitted to the target area via passive transport, upon parenteral administration, the enhanced permeability and retention (EPR) effect has a key role in their accumulation at the target site [57]. Tumor vascular system cut off sizes can vary between 200 and 800 nm. For the micelles to diffuse and accumulate easily into the tumor, their size should be smaller than this limit. Thus, PM size is appropriate for passive targeting, and PMs provide advantages in tumor treatment. A similar situation applies to other diseases characterized by an increased vascular permeability, such as regenerative and autoimmune diseases, inflammation and infection [8]. It is known that vascular permeability increases in these diseases [58,59,60]. The small-sized PMs can easily pass through the vascular endothelial cells and accumulate in the target tissue.

Surface characteristics affect the stability of PMs both in vitro and in vivo as well as their permeation into tissues [61]. For example, PMs with hydrophilic and neutral surfaces are known to increase mucus penetration and have high in vivo stability [62,63]. It is known that positively charged PMs make non-selective protein bonds and show low physical stability [64]. However, their high mucoadhesive properties can positively affect the penetration and permeation of the drug [65]. Another important point of the surface characteristics is that it enables PMs, which are generally transported to the intended area by passive transport, to be actively delivered to the target site by functionalization [57]. The functionalization of PMs is discussed in more detail under the heading Section 2.3. 

Dynamic light scattering (DLS) is the most used method for detecting PM size, PDI and surface charge. Also, atomic force microscopy (AFM), small-angle X-ray scattering (SAXS) and transmission electron microscopy (TEM) can be used to determine both the size and morphology of PMs [61,66].

#### 2.2.2. Physical Stability

Amphiphilic copolymers are found free in low concentrations in aqueous media. As their concentration increases, the system becomes saturated. Increasing the copolymer amount in the medium after the saturation concentration reduces the surface energy of the system. Thus, the copolymers come together to form aggregates. This saturation concentration is called the CMC. When the CMCs of the copolymers forming the micelles are low, the system’s surface energy drops rapidly and becomes stable. In other words, PMs formed with copolymers with a low CMC value are more stable [17].

Fluorescence correlation spectroscopy (FCS), nuclear magnetic resonance (NMR) and Förster resonance energy transfer (FRET) are used to monitor PM structure and stability [8].

#### 2.2.3. Loading Capacity

The determination of the amount of the therapeutic agent in PMs is important; as for effective treatment, the amount of therapeutics in the delivery systems should be high. Two important concepts emerge here: drug loading capacity and encapsulation efficiency. Drug loading capacity is widely defined as the amount of encapsulated drug by the total weight of the system, while encapsulation efficiency describes the percentage of how much of the drug was successfully encapsulated [8]. Loading capacity is greatly influenced by the preparation method of PMs, the polymer used and the polarity of molecules to be loaded [61].

#### 2.2.4. Release of Therapeutic Agent

The determination of the release profile of the therapeutic agent(s) from PMs is important not only as a quality parameter but also because it has critical importance when stimuli-responsive PMs are used in confirming that the system successfully responded to the triggering factor. In these studies, the collected samples containing the therapeutic agent are analyzed by analytical methods such as ultraviolet-visible (UV-Vis) spectrophotometry, high-performance liquid chromatography (HPLC), liquid chromatography–mass spectrometer system (LC-MS) and gas chromatography (GC). By examining the NA loaded in the PM core with an appropriate method in a suitable medium, information about the amount of free NA in the medium against time can be obtained [61]. Based on these findings, the release kinetics of the therapeutic agent from the PM can be determined.

One of the most important factors affecting release from PMs is the interaction between the therapeutic agent and the core. A strong drug–core interaction ensures that the release of the drug from the core is slow. In addition, the proximity of the drug to the polymeric shell facilitates its release from PMs [17].

#### 2.2.5. NA Binding

The interaction between NAs and polymer molecules is an important parameter that should be analyzed for NA-carrying PMs as it affects both release kinetics and the stability of the system. By understanding the interaction that makes up the PIC (as explained in Section 2.1.2), the possible behavior upon the in vivo application of PMs can be predicted. Isothermal titration calorimetry (ITC) is the most common method used to determine NA–polymer binding [67].

### 2.3. Functionalization of PMs

**Figure 4:**
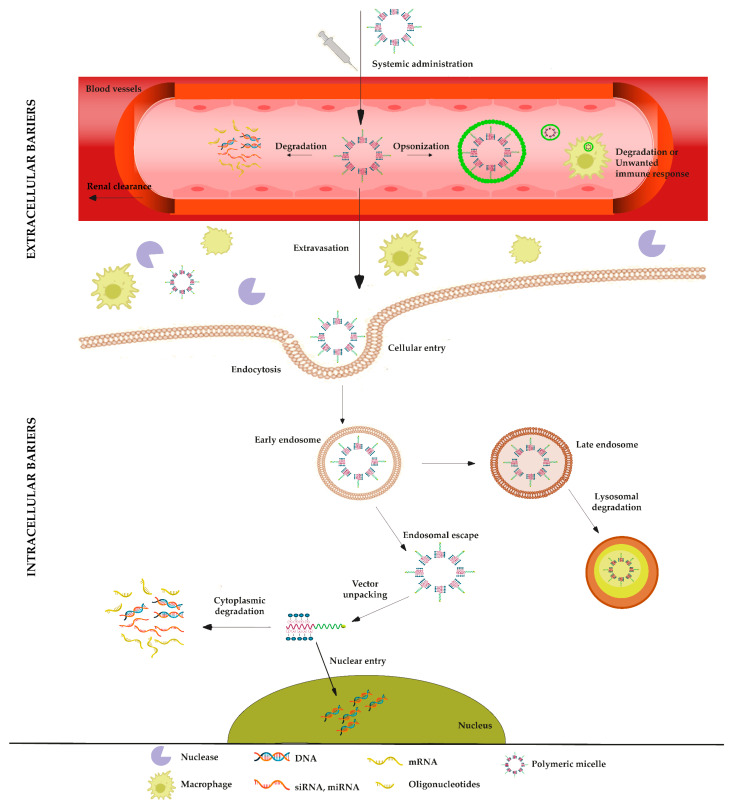
The biological barriers for non-viral gene delivery systems.

As non-viral systems, to be effective gene delivery vectors, PMs should transport NAs to the precise location where a pharmacological effect is expected [68]. During the transportation process, PMs must have the ability to overcome several biological barriers successfully [69]. On the other hand, PMs are expected to protect the NA content from degradation and clearance mechanisms from the moment of application until it reaches the target site. Coating the surface with PEG is the most widely used approach to prepare PMs with long blood circulation time. However, the extracellular and intracellular barriers encountered when NAs are delivered are illustrated in Figure 4 and highlighted in Section 3, where possible solutions are also discussed. By using copolymers with different characteristics, the physicochemical properties of PMs can be controlled to improve the bioavailability and efficacy of NAs. This strategy is known as “functionalization”, and the developed systems are widely named as ‘’smart delivery systems’’.

The different strategies used for the functionalization of PMs are reviewed under this section. The polymers used are given in Section 4.1. 

#### 2.3.1. pH-Responsive PMs

The pH-responsive functionalization of PMs is considered the most common approach, and one of the most useful approaches, for designing PMs with optimal properties [70]. While the pH in normal tissue is 7.4, pH is acidic in the presence of inflammation, tumor tissue, and endosome and lysosome processes [69,71]. Taking advantage of this, it has come to the fore to design PMs that can respond to the change in the pH in different disease conditions and cell processes [57]. In this way, it is ensured that PMs release their content triggered by the target pH.

#### 2.3.2. Reactive Oxygen Species (ROS)-Responsive PMs

Reactive oxygen species (ROS) are byproducts that are produced as a result of enzymatic reactions in various organelles (endoplasmic reticulum and mitochondria) in different cell compartments (e.g., cell membrane and cytoplasm). They are part of basal metabolic function and act as signaling molecules. Thus, they also regulate cellular homeostasis [72].

ROS tend to increase in many diseases, including tumors [73]. For example, while the normal ROS value is 0.02 µM, this value can increase to 100 µM in cancer cells [74]. In such situations, tumor cells increase the production of reducing agents to maintain the balance between ROS production and depletion. Glutathione (GSH) is the main reducing agent in tumor cytosol. The amount of GSH in tumor tissue is about 2–10 mM, which is 100–1000 times higher than in normal tissues [75]. ROS-responsive systems are designed by taking advantage of these differences in GSH expression. Among different designs, the most common strategy is to add a disulfide bond to impart redox sensitivity to PMs.

#### 2.3.3. Enzyme-Responsive PMs

Enzymes are bioactive substances of critical importance, which catalyze substrates in many biological and metabolic processes in the body [75]. The dysregulation of enzyme expression and activity indicates a pathological condition [74]. Changes in enzyme profiles have been associated with many diseases in studies. It has been observed that enzymes that are effective in cell growth, angiogenesis, invasion and metastasis, such as proteases, peptidases and lipases in tumor cells, are much higher than in normal cells [76,77]. Based on this difference, enzyme-responsive PMs can be produced.

#### 2.3.4. Thermo-Responsive PMs

Temperature differences between the human body and its external environment can be considered to develop thermo-responsive delivery systems. For this reason, systems that are activated in situ based on this temperature difference have always been interesting. On the other hand, in cases such as infection, inflammation or tumor presence, tissue temperature rises to higher-than-normal values [78]. Based on this difference, PMs can be specifically designed to initiate drug release at a certain temperature. Temperature-sensitive polymers change phase at a certain temperature known as the low critical solution temperature (LCST) [79]. At a temperature below the LCST, hydrogen bonds form between the water and polymer chains, and the polymers dissolve in water. However, when the LCST is exceeded, the hydrogen bonds are broken and the polymer becomes insoluble, thus triggering the release of NA as the system is active [75].

#### 2.3.5. ATP-Responsive PMs

Adenosine triphosphate (ATP) is the direct energy source of cellular metabolism. A high amount of ATP is needed to counteract the excessive increase in cell growth and angiogenesis in tumor cells. For this reason, the number of mitochondria increases in tumor cells, and they have a much higher value of ATP than normal cells [74]. Based on this ATP difference, ATP-responsive PMs can be developed. For this purpose, the phenylboronic acid (PBA) group and its derivatives are frequently used. PBAs function by selectively forming ester bonds with diol compounds in aqueous solutions.

## 3. Challenges in Delivering Nucleic Acids

For many diseases, the use of nucleic acid constitutes an attractive therapeutic strategy. Although the number of research studies on the potential of DNA- and RNA-based therapeutics has been dramatically expanded, especially during the last decades, and the number of approved nucleic-acid-based products for human use is increasing, their delivery and transport to the site of action remain major obstacles [2]. Overcoming extracellular and intracellular barriers encountered after the administration of these therapeutics could not only accelerate their translation into clinic but also provide treatment for diseases not fully cured. Given that the structure of nucleic acids is considerably different from that of conventional molecules, their stability in biological fluids and delivery to the target cell is a major issue that hinders their efficacy. There are also difficulties related to the site of action that is to be reached for activity; i.e., either the action is to be shown in the cytoplasm or in the nucleus of the cell. However, this point is related to the type and nature of nucleic acid used in therapy, e.g., pDNA, ASO, mRNA, etc. Upon delivery, nucleic acids should be protected and remain stable in physiological fluids and accumulate in target tissue prior to entering the cell. Even though nucleic acids can be delivered via different routes, each route has its own different barriers. Nevertheless, systemic administration remains the main route for delivering DNA- and RNA-based therapeutics [80,81]. The extracellular and intracellular barriers related to the delivery of nucleic acids are discussed below, and the advantages of using PMs in this context are described. The biological barriers for the efficient delivery of systems carrying genetic materials are illustrated in Figure 4.

### 3.1. Extracellular Barriers

One approach for overcoming extracellular barriers, and thus enhancing successful delivery and, consecutively, treatment efficacy, could be the chemical modification of the structure of nucleic acids, which primarily can increase their stability against degradation. Chemical modifications together with conjugation strategies have been extensively reviewed in the literature [82,83,84]. On the other hand, PM-based nanocarriers have shown great potential in overcoming challenges related to gene therapy. They should be designed based on the nucleic acid to be delivered and the delivery route. For efficient transportation at the site of action, stability against nucleases and the avoidance of clearance mechanisms should be provided.

#### 3.1.1. Nuclease Degradation

Nucleic acids are prone to nuclease degradation. Hence, the sensitivity to nucleases of naked nucleic acids requires the use of formulations with special features. When the polyion complex micelles of poly-L-lysine and poly(ethylene glycol) were used as vectors for DNA delivery, it was concluded that negative and positive charge ratios and PLL chain length affected the stability of DNA in blood [85]. While retention time was higher for PIC micelles prepared with charge ratios of 1:4 and a PLL chain length of 48 mer, PIC micelles prepared with shorter PLL chain lengths were less stable in blood [85]. In a more recent study, the capability of anionic dextran-coated micelles to maintain the stability of pDNA in serum was also shown by agarose gel electrophoresis [86]. Nevertheless, the degradation of nucleic acids can be observed even when nanocarriers are used. For this reason, the design of sophisticated nanocarrier formulations is required particularly when mRNAs, which are highly vulnerable to nucleases are delivered [87,88].

#### 3.1.2. Clearance Mechanisms

Renal filtration is vital for the excretion of metabolic waste products from the human body, but nucleic acids with a molecular weight smaller than 30 kDa can be rapidly cleared from the blood by the kidneys, resulting in very short circulation times [89]. On the other hand, larger nucleic acids are rapidly opsonized and cleared via the mononuclear phagocyte system (MPS). It is widely acknowledged that the use of polymeric particles could protect against clearance mechanisms and prolong the time of nucleic acids in circulation. The size of the particle has a critical impact on their biodistribution and pharmacokinetics, and polymeric particles can also be cleared from the bloodstream according to their physicochemical characteristics [90]. PEGylation is a well-established strategy for nucleic acid delivery, which has shown to reduce opsonization and kidney filtration, thus extending circulation and improving therapeutic efficacy. PEG, also known as poly (ethylene oxide) (PEO), is a biocompatible hydrophilic polymer, which is widely utilized to improve the delivery of nanocarriers carrying drugs and genetic payloads. Nevertheless, PEG density, PEG molecular weight, the thickness of the PEG layer formed, etc., are important factors determining the behavior of the delivery system carrying various nucleic acids [91]. Miteva et al. [92] reported that, when micelles prepared with 50 mol% poly(ethylene glycol-*b*-(dimethylaminoethyl methacrylate-*co*-propylacrylic acid-*co*-butyl methacrylate)) and 50 mol% poly(dimethylaminoethyl methacrylate-*b*-(dimethylaminoethyl methacrylate-*co*-propylacrylic acid-*co*-butyl methacrylate)) were administered, micelles prepared with 20 kDa PEG in the corona had a 17.8 min blood circulation half-life versus the 4.6 min that was determined for micelles prepared using 5 kDa PEG. In addition, the increased blood circulation was accompanied by decreased distribution in the kidneys, which could imply a higher stability of micelles prepared with 20 kDa PEG and, as a result, a decrease in the renal clearance of free siRNA [92]. On the other hand, it was found that PEG molecular weight and percentage composition in the corona inversely affected the rate of the cellular internalization of the system. Other studies in the literature also focus on the importance of PEG presence to avoid the clearance of nucleic acid and their delivery systems from circulation and enhance biodistribution after both systemic and local delivery [93,94,95,96]. While an enormous number of studies so far refer to beneficial aspects of using PEG, the clearance mechanism still cannot be completely suppressed. Despite the fact that there is a considerable number of PEG-containing products already authorized for clinical use, issues with its immunogenic potential exist [97].

In addition to PEG, other hydrophilic compounds such as elastin-like polypeptides [98], hyaluronic acid [99,100], poly(*N*-vinylpyrrolidone) and poly(2-oxazoline) [101] have been utilized in the structure of polymeric carriers for improving their in vivo biodistribution.

### 3.2. Intracellular Barriers

After overcoming extracellular barriers and reaching the target site, nucleic acids should efficiently enter the cell. The cellular uptake of non-viral particulate delivery systems commonly occurs via endocytosis. Typically, endocytosis involves the invagination of the plasma membrane that leads to the formation of endocytic vesicles, which transport materials from the extracellular matrix into the cells. Endocytosis is thus the first key process that should occur for PMs to enter the cell [102]. Before reaching the intracellular target, either the nucleus for DNA or the cell cytosol for RNA, PMs containing nucleic acids should pass various intracellular obstacles, which are summarized below.

#### 3.2.1. Cellular Uptake

Several endocytosis pathways have been explored for the entry of outside content into the cell [103]. For nucleic acids, it has been documented that the endocytic pathway affects the transfection efficiency [104]. Endocytosis mechanisms depend on the cell type. Moreover, the physicochemical properties of nanocarriers such as size, shape, charge, functionalization, rigidity and lipophilicity have also been shown to influence cellular uptake [105]. Size-dependent endocytosis has been reported commonly for clathrin- and caveolae-mediated endocytosis. Different studies indicate that particles smaller than 60 nm generally opt for caveolae-mediated endocytosis. Nevertheless, varying results present in the published literature emphasize the role of the different factors mentioned above on the type of endocytosis [106,107,108,109].

For example, negatively charged hyaluronic acid-poly(d,l-lactide-*co*-glycolide)-poly(ethylene glycol) micelles encapsulating 1,2-dioleoyl-3-trimethylammonium-propane (DOTAP)/pDNA lipoplexes demonstrated combined caveolin-mediated endocytosis with macropinocytosis in MDA-MB-231 cells [110], while, in another study, anionic dextran-coated PEI-PLA micelles incorporating an aptamer as a targeting ligand were taken by nucleolin-mediated endocytosis [86]. Taking advantage of this, surface functionalization with specific molecules could be considered to promote specific endocytosis pathways, which could improve the therapeutic efficacy of NAs. Among the targeting ligands explored to facilitate the cellular uptake of polymeric micelles are folate [111,112], iron transport protein transferrin [113] and apolipoprotein E [114]. In a research study published recently, glycyrrhizic acid was integrated in cholesterol-conjugated histidine- and arginine-grafted polyamidoamine micelles (PamHRchol/GA) to increase the cellular uptake of the dendrimer micelles possibly by the binding of glycyrrhizic acid to cellular receptors [115]. The authors in this study reported that glycyrrhizic acid improved the cellular uptake compared to dendrimer micelles without glycyrrhizic acid. Moreover, the results of the study indicated that PamHRchol/GA micelles carrying the heme oxygenase-1 (HO-1) gene provide a useful anti-inflammatory therapy for acute lung injury [115].

#### 3.2.2. Endosomal Escape

Following endocytosis, the particulate carriers, including PMs, carrying NAs are found in endosomes and still physically separated from the cytosol. Endosomal vesicles go through maturation processes during which they are transformed from early endosomes to late endosomes and lysosomes, and the pH inside the vesicles rapidly decreases to 4.5–5 [116]. Thus, it is crucial for nucleic acids to escape endosomes before they are degraded in lysosomes or go through endosome recycling. Endosomal escape is not only affected by the type of endocytosis but also by the nature of the delivery vector. Numerous mechanisms have been proposed in the literature to explain endosomal escape, among which particle swelling, membrane destabilization and proton sponge mechanism are the widely described escape strategies [117]. Particularly, the so-called “proton sponge model” is widely linked with the endosomal escape of PMs [118]. Proton sponge-triggered endosomal escape is mediated by the buffering capacity of polymers because of protonation. As a result, an increase in the osmotic pressure induces the rupture of endosomes and the release of the nucleic acid cargo into cytosol. However, the buffering capacity of the polymers varies, resulting in reduced or absent endosomal escape. Supporting the “proton sponge” concept, PEI has been observed to exhibit high buffering properties, whereas other polymers such as PLL without modification cannot trigger endosomal escape via the proton sponge effect [119]. A more recent study that visualized the endosomal escape of PM carrying siRNA provided further evidence that the proton sponge effect is influenced by PM characteristics [120]. The authors reported that the cytosolic release of siRNA mediated by the proton sponge effect is influenced by the architecture and rigidity of cationic polymer used [120]. In another study, glycolipid-like (galactosylated chitosan-oligosaccharide-SS-octadecylamine) PMs delivering DNAzymes for the gene therapy of hepatitis B showed effective endosomal escape because of chitosan oligosaccharides when their intracellular distribution in HepG2.2.15 cells was determined. The results indicated that DNAzyme delivery via these PMs could be an alternative to treat hepatitis B [121]. Strategies to facilitate endosomal escape have been given in more detail in other specific reviews in the literature [122,123]. Nevertheless, escape from endosomes continues to be a major hurdle that limits the efficacy of NAs, even though considerable research has been carried in the last decades to enhance endosomal escape mechanisms.

#### 3.2.3. Vector Unpacking

For efficient transfection, the nucleic acid should be released from the delivery vector. That is, PMs should be designed, on one side, to allow sufficient interaction with NAs to ensure the stability of NAs and aid their translocation into the cell and, on the other side, to allow their dissociation from the vector so that NAs can interact with the intracellular target site. For DNA delivery, it was reported that vector unpacking is a major limiting step for receptor-mediated PM gene delivery [124]. The vector unpacking of NAs is widely reported to take place in cytosol, although unpacking in the nucleus or endosome is possible [124]. Correspondingly, it is reported that cationized dextran and pullulan modified with the diethyl aminoethyl methacrylate nanoplexes of a p53 plasmid, which were 120 nm in size and had positive zeta potential when prepared at a 5:1 weight ratio, demonstrated vector unpacking in cytoplasm, while DNA was observed to enter the nucleus alone in C6 and HeLa cells [125]. The authors attributed the vector unpacking process to the interaction between the polymer and anionic components present in cytosol. Chen et al. [126] visualized the intracellular unpacking of DNA from polyplexes by quantum dot-FRET. The authors showed that unpacking kinetics correlated with transfection efficiency, which was affected by the type of cationic polymer used. A quantitative comparison of unpacking kinetics suggested that PEI dissociated more rapidly compared to chitosan and polyphosphoramidate. Also, a much higher transfection efficiency for PEI was observed [126]. These results provided evidence on the effect of polymer on intracellular trafficking for the design of effective carriers for gene delivery. Several approaches ranging from the optimization of physicochemical properties of polymers to the development of stimuli-responsive PMs have been designed to facilitate the intracellular release of NA cargo at the appropriate time [127].

#### 3.2.4. Intracellular Transport

Once the NA has been dissociated from the PMs, it should interact with the intracellular target, which can be either in cytosol for RNAs (e.g., mRNA and siRNA) or the nucleus for DNAs (e.g., pDNA). Regardless of the target site, after dissociation from the PMs, NAs will be present in their free form in the cytoplasm where they can be prone to further degradation, which can decrease their half-life dramatically [128]. It should also be mentioned that larger nucleic acid constructs appear to be more susceptible to cytoplasmic nucleases. For example, 40 mer phosphodiester oligonucleotides rapidly degraded, whereas the degradation of 20 mer phosphodiester oligonucleotides appeared to take place more slowly in Vero cells when visualized by fluorescence resonance energy transfer–fluorescence correlation spectroscopy (FRET-FCS) [129]. Moreover, DNA should reach the cell nucleus, which is an additional problem for successful gene expression [128]. PM-mediated gene delivery shows potential to increase stability against cytoplasmic nucleases, as well as to enhance the cytoplasmic mobility and nuclear entry of pDNA.

## 4. Polymeric Micelles for Nucleic Acid Delivery

### 4.1. Polymers and Modifying Agents in Polymeric Micelles

The most critical point in the preparation of PMs with optimal characteristics is the selection of the right polymers and modifying agents. Many characteristic features of the PMs discussed above are directly dependent on the properties and structure of the polymer/modifying agent [13].

There are various types of polymers and modifying agents investigated for the preparation of NA-carrying PMs in studies present in the literature. Table 1 and Table 2 summarize these studies, including the polymers, the type of nucleic acid that is delivered, their application, and the characteristics of the polymer or modifying agent or the type of stimuli-responsiveness.

### 4.2. Plasmid DNA (pDNA) Delivery

Plasmids are extrachromosomal, circular double-stranded DNA molecules that are naturally present mainly in bacteria, but they can also be found in other microorganisms. Plasmid DNA (pDNA) can be genetically engineered to carry genes for encoding specific proteins. They are used for gene therapy to treat or cure several diseases including genetic disorders and cancer, as well as for vaccination [185]. The introduction of therapeutic genes into the cell nucleus can modify gene expression by replacing, inactivating or introducing a particular gene. pDNA is easy to produce, but its delivery to the target cell is still one of the biggest challenges for gene therapy because of rapid enzymatic degradation upon administration and poor transfection, particularly in nondividing cells, which results in limited efficacy [186]. Although pDNA can be delivered simply in its free form as naked DNA, PM-mediated pDNA delivery can provide DNA condensation and protection from degradation, promote both cellular uptake and nuclear delivery, and target release.

It was reported in the early 1990s that the incorporation of plasmids into soluble interpolyelectrolyte complexes, which formed spontaneously due to the electrostatic interaction between DNA and quaternized poly (4-vinylpyridines), enhanced DNA penetration into the cell and in vitro cell transforming efficiency [187,188]. Since then, the understanding of pDNA delivery and transfection efficacy has increased, and different types of cationic polymers have been investigated for the preparation of PMs incorporating pDNA. Among others, PEI, PLL, PAMAM, poly(methacrylate) and chitosan-based micelles have been widely investigated for gene therapy. In this part, the PMs of various polymers that have been explored for the delivery of pDNA are discussed.

PEI is an organic cationic polymer that can be either in linear or branched forms. It is one of the earliest and most studied polymers and has been successfully used for the delivery of pDNA. It is often referred to as the “gold standard” for non-viral vectors due to its high transfection efficacy [189]. However, the molecular weight and structure of PEI affects the performance of PMs. Despite showing better stability and transfection activity, high-molecular-weight PEI induces greater cytotoxicity, whereas PEI with a low molecular weight shows lower transfection activity even though it is more cytocompatible [190]. Recognizing this, modifying PEI with different polymers and groups has been intensely investigated to prolong its systemic circulation and overcome toxicity, aggregation, precipitation and stability limitations when used as polycation transfectant. Among others, grafting PEI with PEG of different molecular weight and structures is extensively studied to enhance the in vitro and in vivo gene expression. Nevertheless, an optimal degree of PEG grafting is essential [191]. Velluto et al. [192] synthesized a triblock copolymer, poly(ethylene glycol)-*b*-poly(propylene sulfide)-*b*-poly(ethylene imine) (PEG-b-PPS-*b*-PEI), and reported that PEG-*b*-PPS-*b*-PEI micelles and the PEG-*b*-PPS/PEG-*b*-PPS-*b*-PEI micelle demonstrated good transfection of pDNA in tumor cells in vitro and in vivo after intratumoral injections while showing markedly reduced cytotoxicity compared to that of linear PEI alone, 10 kDA. Recently, Abd Elhameed et al. [193] showed that cancer cells were efficiently transfected by high-molecular-weight-PEI-based water-soluble lipopolymer containing EGFP-encoding plasmids. While both the investigated dose and cell line affected the toxicity, significant toxicity was not observed at concentrations as high as ≈150 ng per well in A549 and HeLa cells [193]. On the other hand, the modification of low-molecular-weight PEI with either α-tocopherol, cholesterol or diosgenin showed that the polymers with lipophilic parts could form micelles and demonstrated higher transfection efficacy compared to 25-kDa PEI [194].

In another study, pEGFP-C3 plasmid DNA was successfully condensed in polymeric micelles prepared with partially hydrolyzed poly(2-ethyl-2-oxazoline)-*co*-poly(ethyleneimine)-*block*-poly(*ε*-caprolactone), which showed a low CMC, good serum stability and high transfection efficacy of MCF-7 and MDAMB-468 cells [195]. In a more recent study, the potential of magnetic polymeric micelles for targeted drug delivery both for diagnosis and therapeutic purposes in MCF-7 cells was also investigated [196]. Spherical FePECLEFE/DNA micelles (Fe_3_O_4_-PEI-polycaprolactone (FePEC)/folic acid (FA)- polyethylene glycol (PEG)- polyethyleneimine (PEI)-polylactic acid (PLA) (FA-PEG-PEI-PLA-PEI-PEG-FA) (PLEEF)/EPPT peptide (FePECLEFE) micelles) with a particle size of about 200–300 nm not only had good biocompatibility but also showed a high ability to neutralize DNA and protect it against restriction enzymes, resulting in high gene transfer efficiency. In addition, flow cytometry results revealed that micelles prepared at a 10:1:0.5:1 FePEC/PLEEF/EPPT/DNA mass ratio (w/w/w/w %) had the highest gene transfer efficiency in MCF-7 cells in serum-containing and serum-free media. It was also emphasized that, when micelles containing folic acid, which is known to be absorbed into cancer cells by binding to folic acid receptors and endocytosis, were used, the gene transfer efficiency of pEGFP-N1 increased, making these polymeric micelles particularly attractive for use as a theragnostic [196]. In another study, Garg et al. [197] reported the design and synthesis of an amphiphilic cationic polymer–peptide conjugate from a low-molecular-weight PEI (1.8 kDa) and a synthetic peptide, which self-assembled to form positively charged micelles of ~144–205 nm, which varied according to the amount of peptide used. They were cytocompatible while showing comparable transfection of HEK 293 cells with Lipofectamine/pDNA complexes [197].

Poly-L-lysine is one of the poly(amino acids) widely investigated for gene therapy because it is not only positively charged but also contains many active side chain groups. For example, by the reaction of the carboxylic terminal end group of PLGA and the amine group in PLL, amphiphilic graft copolymer PLGA-grafted PLL were synthesized. The preparation of PLGA-grafted PLL micelle/DNA complexes with sizes between 200 and 300 nm and with a positive surface charge demonstrated a transfection efficiency that was about 10-fold higher for pRSVLuc compared to PLL while showing five times lower cytotoxicity, which was probably due to the lower charge density of the PLL micelles [198]. On the other hand, when two cysteines were separately allocated in PEG−oligolysines with 15 or 20 amino acid and cross-linked PMs incorporating luciferase-coding pDNA were formulated, a relationship between the peptide sequence and in vitro gene expression was demonstrated. Although high gene expressions were seen for both PEG−oligolysines with 15 or 20 amino acids in cell-free assays, only micelles containing 20 amino acids showed significant expressions in the cell-based assay in HeLa cells. Additionally, a cysteine addition was required for the stabilization of PEG−peptide PMs via disulfide crosslinks [199]. Another study also described the crucial role of disulfide crosslinking into the poly(ethylene glycol)-*b*-poly(l-lysine) micelle cores to increase the stability of micelles loaded with pDNA encoding an anti-angiogenic protein (sFlt-1) against shear stress in the blood stream and improve their in vivo blood circulation [200].

Polymeric micelles prepared with PAMAM dendrimers have also been proven to be efficient gene transfection vectors for pDNA. While PAMAMs of low generations (G < 3) are easy to synthesize and less toxic in contrast to PAMAMs of high generations (G > 5), their low transfection efficacy does not provide adequate treatment. Tuning the structure of PAMAMs by introducing different groups and altering the physicochemical properties of polymers has been reported to improve the performance of PAMAM-based dendrimers in vitro and in vivo in different studies [201,202]. Piao et al. [203] added a RAGE-antagonist peptide (RAP) to dexamethasone-conjugated polyamidoamine G2 (PAM-D) with the aim of facilitating the PM-mediated intracellular delivery of an APN plasmid for the treatment of acute lung injury. As expected, it was reported that pAPN was successfully delivered in vitro into the L2 cells and that pAPN/PAM-D/RAP had high therapeutic effects in an acute lung injury mouse model, which was attributed to the synergistic effects of RAP and PAM-D. In another study [204], the authors demonstrated that the combined delivery of curcumin loaded into cholesterol-conjugated polyamidoamine PMs and further complexed with the heme oxygenase-1 gene improved gene delivery efficiency and showed greater anti-inflammatory effects in lungs compared to curcumin or plasmid heme oxygenase-1 alone. It was reported that curcumin, which is a hydrophobic drug, can be loaded into the core of the micelles, whereas the plasmid can be complexed via the positive charge on the surface of the micelle [204]. The functionalization of cholesterol-conjugated histidine- and arginine-grafted polyamidoamine PMs with glycyrrhizic acid also could be promising for gene therapy for inflammatory lung diseases [115].

Other PM-based delivery systems, such as T7-conjugated redox-sensitive amphiphilic micelles using polyethylene glycol-polyethyleneimine-poly(caprolactone)-S-S-poly(caprolactone)-polyethyleneimine-polyethylene glycol, which are used to treat breast cancer [205], and chitosan-based micelles for enhanced cellular immunity [206], have also been designed, and their potential to deliver pDNA has been investigated. 

### 4.3. Messenger RNA (mRNA) Delivery

The use of mRNA for preventing or treating numerous diseases has emerged as a promising strategy for several decades. Apparently, the introduction of mRNA-based vaccines in clinics in late 2020 has accelerated even more the development of mRNA therapeutics. Basically, mRNA delivers genetic information into cells where it is translated into a functional protein in cytoplasm [207]. Despite their potential to treat challenging diseases, ease of production, scalability and lack of potential risk of integration with the natural host genome, mRNAs are rapidly degraded by nucleases and show low stability and poor cellular uptake. For successful translation to occur, mRNA should reach the cellular machinery intact. Apart from the rational design of mRNA sequences to increase stability and enhance translational efficiency, different delivery vehicles are being extensively investigated. Even though cationic lipids have been in the spotlight of extensive research to effectively protect and transport mRNA to cells, great interest has been placed on the use of polymers as versatile agents with tunable properties for safe and efficient delivery [208].

The use of polymeric micelles as delivery vectors for mRNA has shown promising outcomes both in vitro and in mouse models against various diseases including neurological disorders, cancer immunotherapy and vaccination [209,210,211,212]. To achieve maximum protein expression, the modulation of micelle properties by different approaches has attracted various investigations. While PEI polymers have been commonly explored for the delivery of mRNA too, its wide therapeutic application remains challenging due to its toxicity, as mentioned previously. Other frequently studied polymers for mRNA delivery are polymethacrylates [213], amino-polyesters, PLL and PAMAM dendrimers [214].

Among other methods, the incorporation of hydrophilic segments, usually PEG chains, into the structure of polymers has been shown to be beneficial for reducing unwanted responses and increasing circulation time and therapeutic efficacy. PEGylated PM nanomicelles of 24 and 34 nm containing four repeating units of aminoethylene groups appeared to elicit low levels of proinflammatory cytokines following intracerebroventicular delivery while providing mRNA protection in a mouse model [215]. However, PEG shielding should be optimized [211]. PEGylated PM demonstrated successful in vivo genome editing in mouse brains. In the corresponding study, Cas9 mRNA and sgRNA of 4.5 kb and 0.1 kb, respectively, were co-delivered using poly(*N*’-(*N-*(2-aminoethyl)-2-aminoethyl) aspartamide as the polycationic segment, emphasizing that PEG was required for effective genome editing [95]. mRNA nanomicelles prepared with polyethylene glycol-poly(*N*’-(*N*-(2-aminoethyl)-2-aminoethyl)aspartamide) block copolymer have been demonstrated to be attractive options for the treatment of spinal cord injury in a mouse model when brain-derived neurotrophic factor mRNA was delivered [216].

Efforts have also been made to improve outcomes of PM-based delivery for gene therapy via hydrophobic modification for the tuning characteristics of hydrophilic polymers. Modifying PEI (1.8 kDa) with vitamin E succinate, which could form self-assembled micelles with an average size of 144.7 ± 0.76 nm at a 32 N/P ratio for mRNA vaccine delivery, resulted in the successful transfection of HeLa, HEK-293T, Vero and DC2.4 cells while exhibiting much lower cytotoxicity compared to the positive control PEI 25k [210]. Also, the synthesis of a stearic acid–PEI copolymer and the formation of self-assembled cationic nanomicelles were shown to improve anti-HIV1 gag-specific immune responses when in vitro transcribed gag mRNA was delivered [217].

Another approach used to enhance the protein expression of mRNA-loaded polymeric micelles both in vitro and in vivo is the use of stimuli-responsive micelles. For this purpose, the introduction of degradable bonds, such as ester and disulfide, is an attractive approach. Yang et al. designed pH sensitive cross-linked micelles of *cis*-aconitic anhydride-modified poly(ethylene glycol)-poly (L-lysine) (PEG-PLL(CAA)) block copolymers that were capable of releasing mRNA triggered by endosomal pH (pH 5.5–4.5) while remaining stable at physiological pH and protecting mRNA from enzymatic degradation [209].

More recently, polymeric micelles based on block copolymer poly(ethylene glycol)-poly(glycerol) (PEG-PG) modified with either glycine (Gly), leucine (Leu) or tyrosine (Tyr) by the formation of ester bonds were investigated for in vivo delivery. In particular, PEG-PG modified with Tyr provided excellent mRNA protection in serum and higher cellular uptake in Huh7 cells. Moreover, mRNA integrity in blood was prolonged after i.v. administration compared to Gly- and Leu-modified micelles. Additionally, when micelles containing firefly luciferase mRNA were evaluated, strong bioluminescent signals were observed making PEG-PGTyr micelles attractive carriers for mRNA delivery [218].

While extensive research in the literature report the delivery of mRNA by local or parenteral injection, current research studies are also being directed towards the administration of mRNA via non-invasive routes. In this content, the inhalable mRNA PMs of hyperbranched poly(beta amino esters) showed localized delivery to the lungs without demonstrating local or systemic toxicity after repeated administrations [219].

The delivery of mRNA using micelles based on cationic lipids and diblock polymers has also been reported to show promising results. For the treatment of colorectal cancer, the biodegradable micelles of DOTAP-poly(ethylene glycol)–poly(*ε*-caprolactone) with a size of 30 nm could efficiently delivery mRNA on C26 mouse colon cancer cells (60.59%) and were effective and safe following systemic administration [220]. Lately, the delivery of mRNA using a combination of lipid- and polymer-based nanoparticles has been explored as an attractive alternative for ornithine transcarbamylase deficiency. When mRNA encoding for ornithine transcarbamoylase (OTC) was administered to mice by the i.v. route, efficient protein production was observed in liver, suggesting that, while lipid nanoparticles protect the mRNA from nucleases, di-block PMs provide specific targeting to the liver and promote the endosomal release of mRNA [221].

It can be concluded that, although most of the non-viral delivery vehicles that are currently in clinical trials investigating mRNA delivery particularly for cancer immunotherapy and vaccination are primarily based on lipid particles, rationally designed polymeric micelles also seem to be attractive candidates that can effectively protect and transport mRNA to cells.

### 4.4. Antisense Oligonucleotide (ASO) Delivery

Antisense oligonucleotides, shortly abbreviated as ASOs, are short, synthetic, single-stranded nucleic acids that can recognize and bind to a specific mRNA, thus modulating gene expression. Despite the increasing interest in ASO therapies, particularly for the treatment of genetic diseases, obstacles to efficient delivery to the cell remain to be overcome. While approaches such as the chemical modification of ASOs are based on the chemical modification of the structure to maintain stability and functionality as they reach cytoplasm, the use of PM-based systems can protect ASOs in the bloodstream and provide opportunity for manipulating delivery at the target site, simultaneously.

Various studies report the incorporation of ASOs in polymeric micelles that have been stabilized via different approaches. For example, Kakizawa et al. [222] synthesized glutathione-sensitive thiolated poly(ethylene glycol)-*block*-poly(l-lysine) that formed micelles crosslinked by disulfide bonds in their inner core, which not only enhanced the stability of the entrapped antisense sequence for vascular endothelial growth factor against nuclease but also improved its intracellular delivery. As an alternative to disulfide crosslinks, polyion complex micelles prepared with triblock copolymers composed of parts with different unique characteristics in terms of hydrophilicity–hydrophobicity–cationic charge can also be utilized for systemic ASO delivery to solid tumors [223]. Notably, the polymer architecture and the presence of cationic moieties in polymers capable of forming PMs play a crucial role for ASO delivery [224].

The use of ASO-loaded micelles could also be promising for systemic brain delivery, despite the presence of the blood–brain barrier (BBB) preventing the penetration of free molecules. The development of glucose-modified polyion complex micelles from poly(ethylene glycol)-*b*-poly(l-lysine) modified with 3-mercaptopropyl amidine and 2-thiolaneimine block copolymers with a size smaller than 50 nm showed efficient accumulation in the brain [225]. Glucose presence could aid the active translocation of the nanocarrier in the BBB via glucose-transporter 1 (GLUT1). ASO-loaded PM-based formulations demonstrated half-lives of 80–100 min in blood circulation compared to 9 min for naked ASO regardless of glucose numbers in their structure. Also, they showed enhanced cellular uptake. Nevertheless, the glucose number was shown to affect MALAT1 knockdown efficiency, with the highest efficiency obtained when 52 glucose molecules were used [225].

Furthermore, ASO–polymer conjugate micelles have emerged in the literature demonstrating distinctive characteristics for gene silencing. Fakhoury et al. [226] showed that when novel ASO–polymer conjugates able to associate into micelles and further complexed with 25 kDa linear PEI (HE_12_-Luc-ASO) were used, significant firefly luciferase knockdown activity was successfully achieved. On the contrary, when ASO was conjugated to a polymer of similar length but with hexaethylene glycol-dodecane units (HE-HEG)_6_-Luc-ASO) that did not form micelles, adequate gene silencing activity was not observed. Herein, using low concentrations of PEI, it was possible to obtain significant transfection and gene knockdown, maintaining minimal cytotoxicity [226].

A similar approach for the intratracheal delivery of ASO to lung cancer was reported recently. When thermoresponsive poly(2-n-propyl-2-oxazoline) of 30k was conjugated with ASO to target taurine-upregulated gene 1 long noncoding RNA (TUG1 lncRNA), a gene which is frequently overexpressed in lung cancers, structures as small as 50 nm with a narrow size distribution (PDI:0.08) were formed. These conjugates reduced the expression level of TUG1 lncRNA at around 55% in the tumor at a dose of 15 µg. Interestingly, significant knockdown activity was not observed for non-conjugated ASO even though it was reported that considerable accumulation was observed in the lung and tumor [227].

Apart from therapeutic applications, the potential of theragnostic micelles co-delivering siRNA/ASO for neural stem cell (NSC) therapy for ischemic stroke has shown encouraging results [228]. Regardless of the benefits NCS treatments seem to offer in clinic, the differentiation of exogenous NSCs into neurons is limited [229]. Hence, *Pnky* lncRNA silencing, which appears to act as an inhibitor of the neuronal differentiation of NSCs, could enhance NSC-based therapy for stroke. MRI-visible nanocarriers composed of a cationic amphipathic polymer (PAsp(DMA)-Lys-(CA)_2_) based on aspartate, lysine and cholic acid and superparamagnetic iron oxide nanoparticles (SPIO) self-assembled into cationic micelles loaded with siRNA/ASO at an optimum N/P ratio of 7/1 showed up to 95.88% in vitro transfection efficiency. Also, 63.2% *Pnky* knockdown efficiency was observed at 24 h after transfection. In addition, an in vivo histological analysis demonstrated that a 5.5-fold increase in neuronal differentiation was achieved for *Pnky*-targeted siRNA/ASO-loaded micelles 2 weeks after intracerebral transplantation in vivo in mice [228].

All the successful outcomes obtained from the studies involving the use of polymeric micelle-based delivery address the beneficial aspects of these systems for the clinical translation of ASO therapies.

### 4.5. Small Interfering RNA (siRNA) Delivery

Small interfering RNAs (siRNA) are the cleavage products of dsRNA that can induce the deliberate silencing of protein coding genes. They are also named silencing RNA or short interfering RNA. The use of synthetic siRNA therapeutics for challenging diseases, particularly for various types of cancers and genetic diseases, has been a fast-growing area of research following the introduction of the RNA interference (RNAi) concept for gene silencing in early 1998 and the approval of the first siRNA therapeutic (patisiran) in 2011 for an orphan disease, hereditary transthyretin (hATTR) amyloidosis [230]. Polymers generally investigated for gene delivery are also studied for the development of PMs for siRNA. Some of the most explored synthetic polymers since early studies are PEI [231,232], PLL [233], poly(amido amine) [234,235] and PCL [137,149]. Furthermore, pH-responsive polymers are also widely explored for the rational design of PMs for siRNAs [236,237].

In line with previous studies reporting the successful delivery of nucleic acids (i.e., pDNA) by the lipid modification of PMs, the delivery of siRNA via lipid (DOTAP)-modified monomethoxy poly(ethylene glycol)-poly(*ε*-caprolactone) (MPEG-PCL) hybrid cationic PMs for colon cancer therapy was efficacious. Anticancer activity seen in vitro could be due to the inhibition of the proliferation of C26 cells. Also, gene silencing was obtained in vivo [137]. In another study, the peptide modification approach was used to prepare CH2R4H2C-peptide-modified MPEG-PCL nanomicelles (~60 nm) delivering the NF-κB-targeting siRNA (siRelA) gene for effective treatment. After i.v. administration in a mice model, anti-inflammatory activity against ulcerative colitis was seen, which was also characterized by a decrease in the shortening of an inflamed large intestine, clinical score and inflammatory cytokine production [238].

PMs could be interesting candidates for synergistic therapy against cancer to co-deliver siRNA with chemotherapeutic small molecule drugs. Li et al. synthesized an amphiphilic block copolymer to prepare a pH-sensitive micelle having PEG in the shell to increase in vivo circulation, PLL to facilitate siRNA loading and poly(aspartyl(benzylamine-co-(diisopropylamino)ethylamine) in the hydrophobic core for enhancing stability and pH sensitivity. Positively charged PMs of a small size (~70 nm) successfully co-delivered siRNA and doxorubicin to mice via tail injection and were accumulated at the tumor site [239]. Conversely, Jiang et al. proposed the use of cation-free self-assembled micelles based on siRNA conjugates linked to poly(*N*-isopropylacrylamide) diblock copolymer via a redox-sensitive disulfide bond. These siRNA micelles displayed effective BBB penetration for the treatment of glioblastoma. Moreover, synergistic therapy was demonstrated in a temozolomide-resistant tumor when the chemotherapeutic drug temozolomide was co-delivered with siRNA micelles to knockdown tumor-associated genes [45].

Although synthetic polymers dominate the field of PM-mediated gene delivery, various natural compounds such as chitosan [240], hyaluronic acid [241] and cyclodextrins [242] are also explored for siRNA delivery. Among others, chitosan offers distinct advantages as a natural polymer to develop non-viral vectors for siRNA. Chitosan is a positively charged non-toxic, biocompatible and biodegradable natural polysaccharide [243]. Self-assembling cholesterol-conjugated chitosan micelles co-delivering siRNA and salinomycin showed improved in vitro cytotoxicity against both SNU-668 and SGC-791 gastric cancer cells and more potent tumor suppression compared to free salinomycin in vivo [244]. Recently, carboxymethyl chitosan, a water-soluble derivative of chitosan, was utilized to prepare multifunctional micelles grafted with an epidermal growth factor receptor (EGFR)-specific ligand, GE11 peptide, for tumor targeting. The co-delivery of doxorubicin with PD-L1 siRNA, which can inhibit PD-L1 expression and reactivate immune responses against malignant cells, using peptide-modified carboxymethyl chitosan micelles enhanced the anti-tumor effect in an orthotopic-tumor-bearing mouse model when administered i.v. [245].

## 5. Conclusions and Future Perspectives

Nucleic acids such as pDNAs, mRNAs, ASOs and siRNAs provide new cutting-edge treatment opportunities at the molecular level for various diseases as well as vaccination. However, their relatively fragile structures face different extracellular and intracellular barriers when delivered, and they can hardly reach the target site of activity. To date, a wide range of non-viral systems have been investigated for gene therapy. In this review, we have addressed the use of polymeric micelles for the delivery of nucleic acids by highlighting the different methods used for their preparation and their characteristics and providing an update on their current status for the delivery of widely investigated DNA- and RNA-based therapeutics. The characteristics of the PMs are very important for ensuring effective and safe treatments.

PMs prepared using polycationic polymers continue to be a significant part of scientific research carried for gene delivery. They are particularly advantageous because of their relatively good safety profile, large-scale production and cost when compared especially with viral delivery systems. While high levels of transfection efficiency have been achieved with a wide range of studied polycationic polymers, polymers such as PEI and PAMAM suffer primarily from inherent cytotoxicity, which is widely attributed to their positive charge. Indeed, charge is an important parameter affecting, amongst other things, the interaction with nucleic acids. Tuning the structural properties of polymers, including charge, molecular weight, hydrophilicity and degradability, and the functionalization of PMs for passive and active targeting may provide high therapeutic benefits while reducing side effects.

It should be noted that the number of nucleic-acid-based products entering clinics is increasing. While significant progress has been made, and various interesting PMs have been described and tested mainly in the preclinical phase, various biological barriers, stability in blood and other biological fluids, biodistribution and targeted delivery remain major challenges for efficient gene therapy. Nevertheless, increasing knowledge on molecular mechanisms, developments in nanotechnology and production processes could lead to the design and development of safe and effective polymeric micelles for various nucleic acids, which could pave the way for not only new but also more effective treatments for a large variety of patient populations.

## Figures and Tables

**Table 1:** Polymers/modifying agents used in the preparation of NA-carrying PMs, the type of NA and the target disease category.

Polymer/Modifying Agent	Characteristic	Nucleic Acid	Disease Category	References
Aliphatic chain	Hydrophobic	siRNA	Oncology	[130]
Amines	Cationic	siRNA	Oncology	[130]
APA	Amphiphilic	siRNA	Oncology	[130]
APD	Cationic	pDNA	Oncology	[131]
Cholic acid	Hydrophobic	pDNA	Oncology	[131]
CCP	Anionic	siRNA	Oncology	[132]
CPP	Cationic	miRNA	Oncology	[133]
		siRNA	Oncology	[134,135]
Cys-bridged His-Arg	Cationic	siRNA	Oncology	[136]
DOTAP	Cationic	siRNA	Oncology	[137]
		pDNA	Oncology	[138]
DP	Cationic	siRNA	Oncology	[139]
EMA	Cationic	siRNA	Oncology	[140]
MAODCA	Hydrophobic	pDNA	Oncology	[141]
NVP	Cationic	mRNA	-	[142]
PAGA	Cationic	siRNA	Oncology	[143]
pAsp	Cationic/Hydrophobic	mRNA	Neurology	[95]
		siRNA	Viral	[144]
		siRNA	Oncology	[134,135,145]
		-	Orthopedia	[146]
PCC	Cationic	miRNA	Oncology	[147]
PCL	Hydrophobic	siRNA	Oncology	[137,139,148,149,150,151,152,153,154,155]
		pDNA	Oncology	[138,156]
pCys	Hydrophobic	miRNA	Oncology	[157]
PDMAEMA	Cationic	siRNA	Oncology	[148,149,155,158]
		pDNA	-	[141,159]
PDPA	Cationic/Hydrophobic	siRNA	Oncology	[143]
		pDNA	Oncology	[159]
PE	Hydrophobic	siRNA	Oncology	[43]
PEG/mPEG/PEO	Hydrophilic	pDNA	Oncology	[138,156,160]
		siRNA	Oncology	[43,134,135,136,137,140,143,145,150,151,152,153,154,155,161,162,163,164,165]
		miRNA	Oncology	[133,147,157]
		mRNA	Neurology	[95]
		-	Orthopedia	[146]
PEI	Cationic	pDNA	Neurology	[147]
		pDNA	Oncology	[156,166]
		siRNA	Oncology	[150,163,167]
PGA	Hydrophobic	siRNA	Oncology	[140]
PHB	Amphiphilic	siRNA	Oncology	[158]
PHis	Hydrophobic			
PLA	Hydrophobic	mRNA	-	[142]
		miRNA	Oncology	[133]
PLGA	Hydrophobic	pDNA	Neurology	[147]
		siRNA	Oncology	[164,167]
PLL	Cationic/Hydrophobic	siRNA	Oncology	[152,164]
		miRNA	Oncology	[157]
		siRNA	Oncology	[145,168]
PMPMC	Hydrophobic	siRNA	Oncology	[161]
P(NAS-co-NVP)	Amphiphilic	mRNA	-	[142]
POEOMA	Hydrophobic	pDNA	Oncology	[159]
Polystyrene	Hydrophobic	siRNA	Oncology	[136]
PPA	Cationic	siRNA	Oncology	[162]
PPEEA	Cationic	siRNA	Oncology	[153,154]
PSMA	Amphiphilic	siRNA	Oncology	[136]
RGD	Amphiphilic	siRNA	Oncology	[168]
SP	Cationic	siRNA	Oncology	[139]
TAT	Cationic	siRNA	Oncology	[151]
TEPA	Hydrophobic	miRNA	Oncology	[147]
TP	Cationic	siRNA	Oncology	[139]
TPP	Hydrophobic	pDNA	Oncology	[166]

APA: γ-aminohexane(40%)-diaminoethane(60%)-L-polyglutamate; APD: assymetric peptide dendrimer; CCP: charge-conversion polymer; CPP: cell-penetrating peptide; DOTAP: dioleoyl-3-trimethylammonium propane; DP: dimethyldipropylenetriamine; EMA: amino-substituted ethyl methacrylate; MAODCA: 2-(methacryloyl)oxyethyl-2-hydroxyethyldisulfide cholate; NVP: *N*-vinylpyrrolidone; PAGA: poly(2-(diisopropylamino)ethyl methacrylate); pASP: poly{*N*’-[*N*-(2-aminoethyl)-2-aminoethyl]aspartamide}; PCC: poly(2-methyl-2-carboxyl-propylene carbonate); PCL: polycaprolactone; pCys: poly(L-cysteine); PDMAEMA: poly(2-dimethylaminoethyl methacrylate); PDPA: poly(2-(diisopropylamino)ethyl methacrylate); PE: phosphatidylethanolamine; PEG: poly(ethylene glycol); mPEG: methoxy poly(ethylene glycol); PEO: poly(ethyleneoxide); PEI: polyethylenimine; PGA: poly(glycidyl methacrylate); PHB: polyhydroxybutyrate; PHis: poly(L-histidine); PLA: poly lactic acid; PLGA: poly(lactic-*co*-glycolic acid); PLL: poly(L-lysine); PMPMC: poly(5-methyl-5-propargyl-1,3-dioxan-2-one; P(NAS-*co*-NVP): poly(*N*-acryloxysuccinimide-*co-N*-vinylpyrrolidone); POEOMA: poly(oligo(ethylene oxide) monomethyl ether methacrylate); PPA: polyphosphoramidate; PPEEA: poly(2-aminoethyl ethylene phosphate); PSMA: poly(styrene-*co*-maleic anhydride); RGD: arginylglycylaspartic acid; SP: spermine; TAT: transactivator of transcription-derived peptide (GRKKRRQRRRPQ); TEPA: tetraethylenepentamine TP: tetraethylenepentamine; TPP: α-tocopherol.

**Table 2:** Stimuli-responsive polymers used in the preparation of PMs and the corresponding type of NA.

Stimuli-Responsiveness	Functional Vector	Nucleic Acid	References
pH-responsive	PLL-polyhistidine	siRNA	[169]
	Poly(styrene-alt-maleic anhydride)	DNA	[170]
	Cross-linked low M_w_ PEI by imine linkers	DNA	[171]
	Lactosylated PEG–PLL	siRNA	[172]
	Ketalized PEI	DNA/siRNA	[173]
ROS-responsive	PEG–thiolated PLL	DNA	[174]
	PEG–thiolated PLL	siRNA	[175]
	PEG–thiolated PLL–melittin–siRNA	siRNA	[176]
	PEI–PHPMA	DNA	[177]
	Cyclodextrins threaded onto PEG	DNA	[178]
Enzyme-responsive	PEI-FPBA/Chol-DOPA	siRNA	[179]
	PEG-PLG^∗^LAGr_9_–PCL	siRNA	[180]
	PEG-pp-PEI-PE	siRNA	[181]
Temperature-responsive	PEI–poly(NIPAM–acrylamide)/PEI–poly(NIPAMvinylpyrrolidone)	DNA	[182]
ATP-responsive	FPBA-functionalized/PEG-PLL	siRNA	[183]
	PEG-PBA	mRNA	[184]

CAT: Chloramphenicol acetyltransferase; Chol: cholesterol; DOPA: dopamine (with diol-containing moiety); FPBA: 3-fluoro-4-carboxyphenylboronic acid; M_w_: molecular weight; NIPAM: *N*-isopropylacryamide; PCL: polycaprolactone; PE: phosphatidiletanolamine; PEG: poly(ethylene glycol); PEI: polyethylenimine; PHPMA: poly(*N*-(2-hydroxypropyl)methacrylamide); PLG^∗^LAGr_9_: Pro–Leu–Gly–Leu–Ala–Gly–Arg–Arg–Arg–Arg–Arg–Arg–Arg–Arg–Arg; PLL: poly(L-lysine); PNIPAAm-*b*-PMMA: poly(*N*-isopropylacrylamide-*b*-methyl methacrylate).

## Data Availability

Not applicable.
